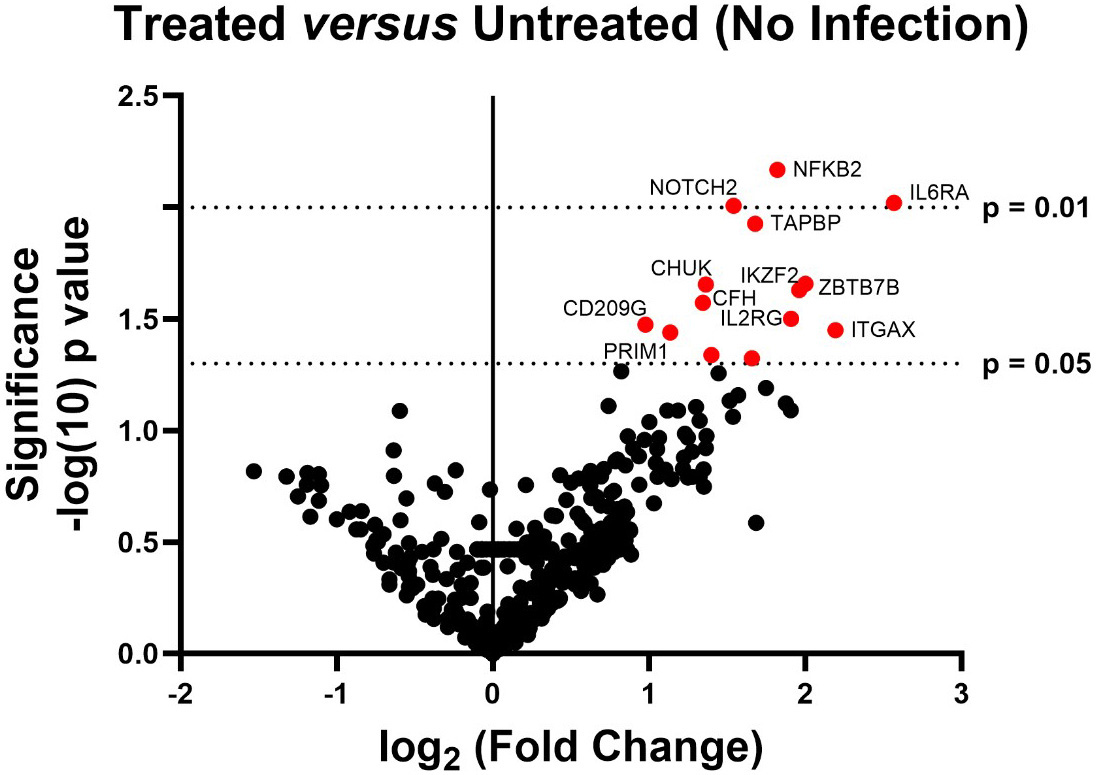# 513 Persistent Cutaneous NO Delivery Did Not Alter Burn Wound Conversion Kinetics but Induced Immune Reprogramming

**DOI:** 10.1093/jbcr/iraf019.142

**Published:** 2025-04-01

**Authors:** Caroline Lunny, Madelyn Bucci, Denise Hernandez, Matthew Warchol, Samantha Picciotti, Quincy Grayton, Heba El-Ahmad, Shannon Wallet, Mark Schoenfisch, Robert Maile

**Affiliations:** University of Florida; University of Florida; University of Florida; University of Florida; University of North Carolina at Chapel Hill; University of North Carolina at Chapel Hill; University of Florida; University of Florida; University of North Carolina at Chapel Hill; University of Florida

## Abstract

**Introduction:**

The burn wound healing process is dysfunctional. Conversion of previously undamaged tissue to damaged can prolong the healing process with increases in morbidity and mortality of patients. Therapies that utilize anti-inflammatory molecules, like nitric oxide (NO), have been proposed to modulate this inflammatory process. In low doses, NO has been shown to downregulate innate immune cells, stimulate angiogenesis, and promote fibroblast activity at the wound site. NO is physiologically unstable on its own. However, when coupled with other matrices, it can be released in a controlled state. Our collaborators utilize an amine (N-(2-hydroxyethyl)ethylenediamine; HEDA)-modified Chondroitin-sulfate C (CSC) scaffold that delivers NO to tissue, with minimal toxicity, and possess non-burn wound regenerative properties. We hypothesize that in combination with pluronic F127 organogel to aid efficient and stable delivery to wound tissue, CSC-HEDA/NO will modulate wound conversion and reduce healing time for burn wounds in mice.

**Methods:**

Wildtype female C57BI/6 mice weighing 18-22g underwent a 20% total body surface area full-thickness cutaneous contact burn or sham injury (n=6) with appropriate anesthesia. Analgesia was provided through the length of the experiment. Mice were treated immediately after injury with 100ul of CSC-HEDA/NO directly applied to the wound surface, and every three days thereafter for 14 days. We evaluated rate of wound closure, grade of inflammation and wound score, and wound conversion. At 14 days, mice were euthanized and wounds were removed for RNA isolation. nanoString immune transcriptomic analyses (Mouse Immunology CodeSet) and corresponding Ingenuity Pathway Analysis (IPA) were performed.

**Results:**

We found no significantly altered conversion between the groups, and observed decreased closure in the CSC-HEDA/NO group at 8, 11, 12, 13 and 14 days post-injury compared to the untreated group. Other wound scores were unchanged between groups. However, transcriptomic analysis showed a significant (P< 0.05) upregulated of 14 immune genes compared to untreated wounds, including IL6RA (+2.3 fold-change (Fc)), NFKB2 (+1.8fc) and ITGAX (+2.1fc). IPA demonstrated significant alteration in canonical immune pathway activation with significant reprogramming (induction) of the IL-4/IL-13, Th1/Th2 activation, and FAK signaling pathways.

**Conclusions:**

Studies of the role of NO in wound healing by multiple groups has demonstrated diverse pro- or anti-inflammatory effects, and these data reveal a significant induction of inflammatory reprogramming in burn wound tissue. Histology data are still pending which will further reveal mechanism of the decreased wound closure. We are currently investigating the use of CSC-HEDA/NO during concurrent bacterial infection.

**Applicability of Research to Practice:**

NO has been suggested in studies to improve burn wound outcomes with often paradoxical outcomes. These data add to the body of knowledge.

**Funding for the Study:**

N/A